# The combined angiographic technique during mechanical thrombectomy for predicting first-pass effect in patients with large artery occlusion: A retrospective observational cohort study

**DOI:** 10.1097/MD.0000000000046581

**Published:** 2025-12-19

**Authors:** Chao Xu, Danyu Chen, Guixing Xu, Shufeng Yu, Tianbo Xu, Jiangxian Ying, Peng Wang

**Affiliations:** a Center for Rehabilitation Medicine, Department of Neurology, Zhejiang Provincial People’s Hospital, Affiliated People’s Hospital, Hangzhou Medical College, Hangzhou, China; b Department of Neurology, Tongxiang First People’s Hospital, Tongxiang, China; c Department of Neurosurgery, The First Affiliated Hospital, Sun Yat-sen University, Guangzhou, China; d Department of Radiology, Zhejiang Provincial People’s Hospital, Affiliated People’s Hospital, Hangzhou Medical College, Hangzhou, China; e Department of Neurology, Tiantai People’s Hospital of Zhejiang Province, Tiantai, China; f Department of Neurology, Taizhou First People’s Hospital, Taizhou, China.

**Keywords:** angiography, first pass effect, large artery occlusion, mechanical thrombectomy, predictors

## Abstract

First pass effect (FPE), defined as single-pass complete or near-complete reperfusion during endovascular thrombectomy for large artery occlusion (LAO) strokes, is a significant performance metric, which is increasingly utilized to determine the success of mechanical thrombectomy (MT). To evaluate the value of the innovatively proposed combined angiographic technique (CAT) for predicting FPE in anterior circulation stroke patients with LAO. We retrospectively reviewed our prospectively collected data for anterior circulation LAO patients treated with MT between January 2019 and December 2022. FPE was defined as single pass of the device, near-complete/complete reperfusion of the LAO and its downstream territory (mTICI 2c/3) after MT, and no use of rescue therapy. The cohort was categorized into the FPE and non-FPE groups. Angiography is performed with an intermediate catheter and micro-catheter, which we refer to as the CAT, to determine the location and length of the thrombus body during the procedure. The sensitivity, specificity, and positive and negative predictive values of the CAT in predicting FPE were assessed. A total of 217 patients with anterior circulation occlusion were included in the final analysis. Among them, combined angiographic technique was conducted in 94 (43.3%) patients. Patients with FPE used the CAT prior to MT at a higher rate than those without FPE (69.2% vs 11.3%, *P* < .001). The sensitivity, specificity, positive predictive value, and negative predictive value of the CAT in predicting FPE were 69.2%, 88.6%, 88.3%, and 69.9%, respectively. Our study showed that the CAT is technically safe and easily applied in clinical practice, which may be helpful for neuro-interventionists to select the appropriate thrombectomy strategy and optimal material combination during the procedure. Further prospective multicenter studies need to validate this safe and efficient technique in a large patient cohort.

## 1. Introduction

Mechanical thrombectomy (MT) is the standard treatment in anterior circulation strokes with large artery occlusion (LAO).^[1,2]^ Although MT has improved functional outcomes for LAO patients, up to 50% of patients still experience significant functional impairment after successful reperfusion.^[3,4]^ Given that establishing reperfusion in a timely manner can limit infarct size and minimize neuron loss, achieving successful recanalization at the fastest possible pace is essential for a favorable outcome.

First pass effect (FPE), defined as a single pass with complete recanalization of the LAO and its downstream territory without rescue therapy, has emerged as a critical predictive metric.^[5,6]^ Although previous studies have demonstrated that FPE is associated with favorable functional outcomes and lower mortality rates,^[7,8]^ definitive predictors of FPE remain elusive. Many factors may affect FPE rates, including histologic thrombus structure, location, etiologies, vessel anatomy, and thrombus burden.^[9–12]^ The most important aspect of FPE is the removal of the thrombus in a single pass. For FPE to be achieved, it is essential to assess the location and length of the thrombus body accurately during the procedure. Because accurately assessing the location and length of the thrombus body during the procedure is crucial for neuro-interventionists to select the appropriate thrombectomy strategy and optimal material combination. Nevertheless, intraprocedural angiographic imaging can only visualize the proximal blind end of the thrombus and cannot determine the distal end of the thrombus. Consequently, neuro-interventionists were often limited to estimating the length and location of the thrombus based on individual experience, resulting in low thrombectomy efficiency, prolonged procedure time, and poor outcomes.

In view of these considerations, we innovatively proposed a combined angiographic technique (CAT), which involves simultaneous digital subtraction angiography through an intermediate catheter and an angiographic microcatheter during the procedure, and the distal end of the thrombus is shown by contrast medium retrograde. It is assumed that FPE may be more likely to be achieved during MT with CAT. Thus, we conducted a retrospective study to evaluate the value of the newly proposed CAT for predicting FPE in anterior circulation stroke patients with LAO.

## 2. Materials and methods

### 2.1. Study subjects

We retrospectively reviewed our prospectively collected database of our single stroke center for consecutive patients with LAO who received MT between January 2019 and December 2022. We enrolled patients who (1) had CT angiography-confirmed LVOs presenting within 6 hours of symptom onset, including internal carotid artery, middle cerebral artery (M1 or M2). Patients presenting at the hospital 6 to 16 hours after symptom onset were included if they met the criteria described in the DEFUSE-3 trial.^[13]^ Patients presenting 6 to 24 hours after symptom onset were included if they met the related criteria described in the DAWN trial.^[14]^ The exclusion criteria were as follows: patients with a baseline Alberta Stroke Program Early CT Score (ASPECTS) < 6; received angioplasty or stent implantation as the first-line technique; tandem occlusion; and angiography images with poor quality.

### 2.2. Date collection and endovascular procedure

We retrieved demographic, clinical, and radiological data, including age, sex, baseline National Institutes of Health Stroke Scale (NIHSS) score, baseline systolic blood pressure and diastolic blood pressure parameters, comorbid conditions such as history of smoking, hypertension, diabetes mellitus, atrial fibrillation, and hyperlipidemia, time from puncture to reperfusion, the site of occlusion, and first-line thrombectomy strategy. In our stroke center, all endovascular procedures were performed under a dedicated neuro-anesthesia protocol that included conscious sedation or general anesthesia. The first choice of MT strategy (stent retriever, contact aspiration, or both) was left to the discretion of the neuro-interventionists. All imaging data in this study were evaluated by 2 neurologists (C.X. and X.J.), blinded to the clinical and procedural data. Discrepancies were reviewed by another experienced neurologist (P.W.) and settled by consensus discussion afterward.

### 2.3. Protocol of the combined angiographic technique

**Figure 1.**
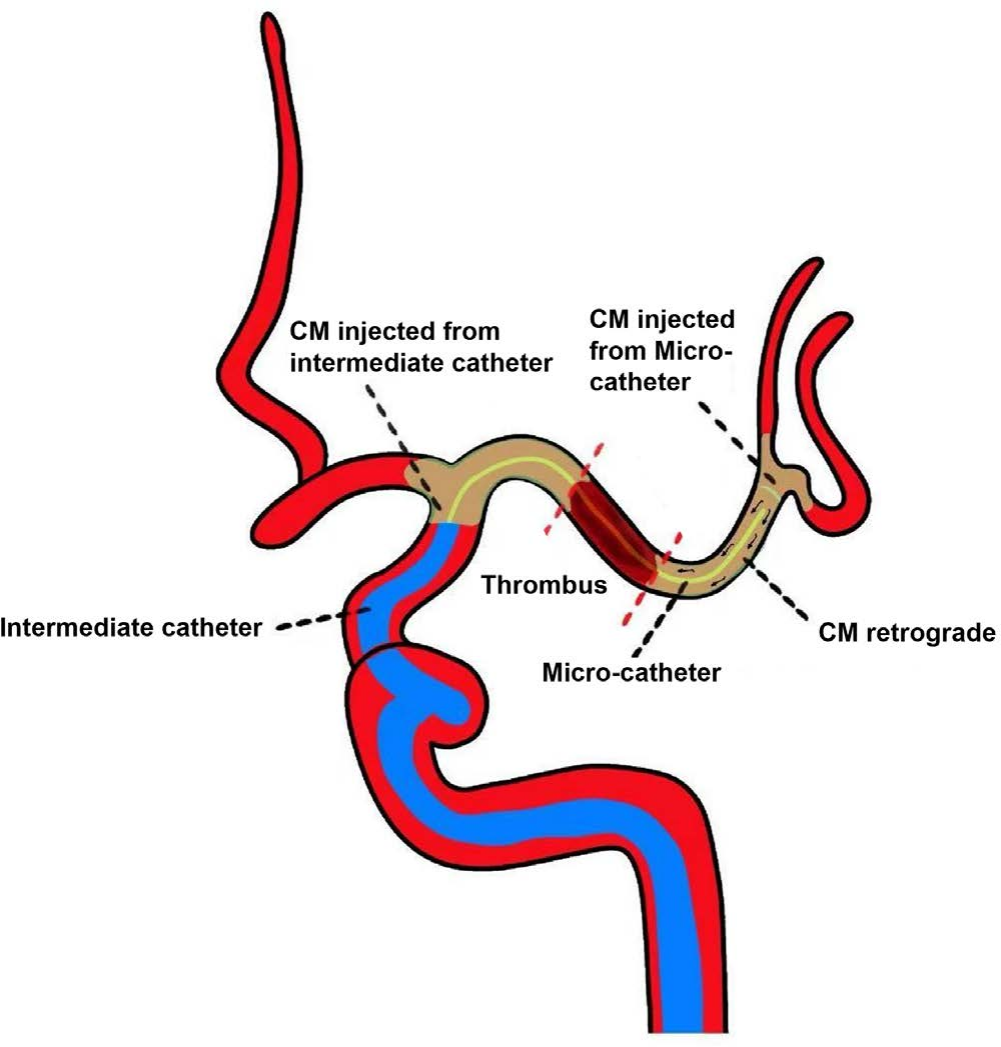
A schematic diagram of the combined angiographic technique. CM = contrast medium.

**Figure 2.**
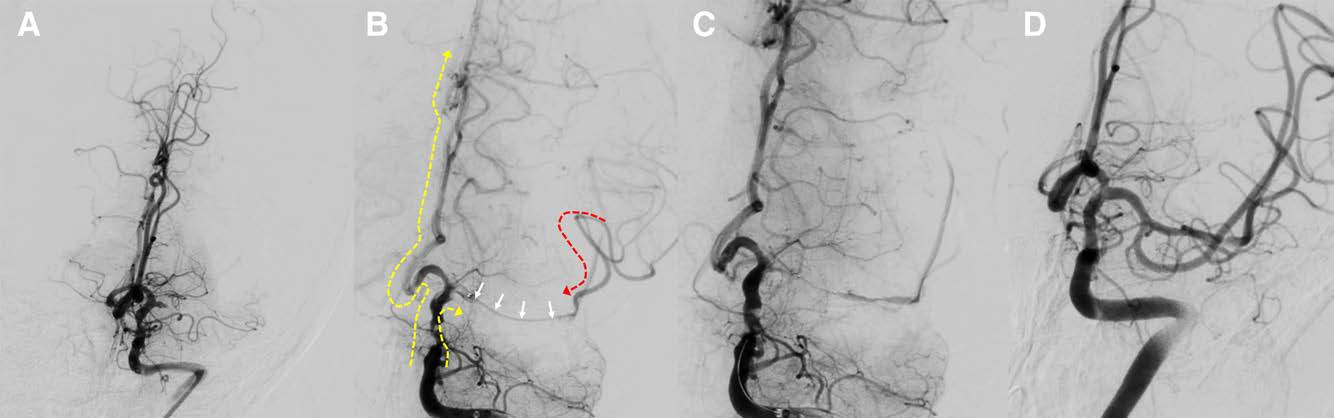
Illustrative case of the combined angiographic technique. A 61 years old male patient presented with right hemiparesis and aphasia. (A) DSA revealed total occlusion of the proximal segment of the left MCA. (B) Angiography is simultaneously performed with an intermediate catheter and micro-catheter. The location and length of the thrombus was clearly observed (white arrow). The distal end of the thrombus is recognizable via retrograde contrast medium (red dashed line). The yellow dashed line indicates the forward contrast agent administered with the intermediate catheter. (C) A 6.0-mm × 30 mm Solitaire stent was unsheathed at the occlusion site. (D) mTICI grade 3 recanalization was achieved after a single pass of the stent retriever. DSA = digital subtraction angiography, MCA = middle cerebral artery, mTICI = modified treatment in cerebral infarction.

The decision to use CAT was mainly based on the discretion of the neuro-interventionists. In addition, in certain instances, such as patients with intracranial atherosclerotic stenosis, if the microcatheter has difficulty passing the lesion or if there is a large burden of thrombus at the internal carotid artery terminus, we will select a large-diameter intermediate catheter for aspiration directly rather than utilizing CAT. A schematic diagram of the combined angiographic technique is shown in Figure 1. First, the optimal working angle was determined, and the proximal end of the thrombus was identified by injecting contrast agent from the intermediate catheter. Once the proximal occlusion position was identified, the Synchro microwire (Stryker Neurovascular, Fremont) was employed to facilitate the navigation of Rebar-18 Microcatheter (Medtronic, Minneapolis) into the portion distal to the occluded site. The microwire is then withdrawn and prepared for angiography. Subsequently, the contrast agent is administered via hand bolus injection simultaneously through both an intermediate catheter and micro-catheter, the surgeon uses a 10 mL syringe to inject 5 to 6 mL of contrast agent from the intermediate catheter, while the assistant uses a 2 mL syringe to gradually push 1 to 2 mL of contrast agent into the microcatheter for a duration of 2 to 3 seconds. The contrast agent administered through the intermediate catheter terminates at the proximal end of the thrombus, while that delivered via the micro-catheter flows retrograde to the distal end of the thrombus. And the distal end of the thrombus is visible by contrast agent retrograde. This technique, referred to as the combined angiographic technique, ascertains the location and length of the thrombus body by devoiding the thrombus of contrast agent. Figure 2 shows the representative case of the combined angiographic technique during MT.

### 2.4. Tips of the combined angiographic technique

The CAT is technically safe and easily applied in clinical practice since it requires no specialized technical training and does not increase additional procedural time and risks. It is worth noting that the successful application of CAT also necessitates the experience level of operators and adequate coordination between the surgeon and the assistant, a potential barrier to widespread adoption of this technique. Prior to CAT, an experienced surgeon may initially inject 1 to 2 mL of the contrast agent into the intermediate catheter. This is attributable to the interior volume of the intermediate catheter compromising a portion of the injected contrast.

In addition, as the CAT was delivered through manual injection, the surgeon might receive direct force feedback during the injection procedure. This feedback could enable the surgeon to adjust the injection power and avert the application of excessive force during the administration of contrast agents. At our center, all surgeons and assistants are established partners with more than 5 years of expertise in MT.

### 2.5. Outcome

The primary outcome was the presence or absence of FPE. FPE was defined as a single pass of the device, near-complete/complete reperfusion of the LAO and its downstream territory (modified treatment in cerebral infarction [mTICI] 2c/3) after MT, and no use of rescue therapy.^[5,6]^ FPE determination was blinded to the use of CAT. The mTICI score is a commonly used scale for documenting the results of revascularization strategies in acute stroke treatment.^[15]^ mTICI was classified by using angiographic criteria as follows: Grade 0, no perfusion. Grade 1, antegrade reperfusion past the initial occlusion, but limited distal branch filling with little or slow distal reperfusion. Grade 2a, antegrade reperfusion of less than half of the occluded target artery previously ischemic territory (e.g., in 1 major division of the middle cerebral artery and its territory). Grade 2b, antegrade reperfusion of more than half of the previously occluded target artery ischemic territory (e.g., in 2 major divisions of the middle cerebral artery and their territories). Grade 2c, near complete perfusion except for slow flow or distal emboli in a few distal cortical vessels. Grade 3, complete antegrade reperfusion of the previously occluded target artery ischemic territory, with absence of visualized occlusion in all distal branches.

### 2.6. Statistical analysis

Study subjects were dichotomized as the FPE group and non-FPE group. Clinical characteristics were summarized as mean ± SD or median (25th–75th percentile) for quantitative variables depending on the normality of the distribution and as frequency (percentage) for categorical variables. Fisher’s exact test was used to compare the dichotomous variables between 2 groups, whereas an independent sample 2-tailed *t* test or a Mann–Whitney *U* test was used for the continuous variables, depending on the normality of the distribution. Associations of variables with FPE were determined using binary logistic regression models adjusted by characteristics with a *P* value of < .1 in univariate analyses, respectively. Receiver operating characteristic curve analysis was used to determine predictive value. All statistical analyses were performed using SPSS, Version 22.0 (IBM, Armonk). A *P* value < .05 was considered statistically significant.

## 3. Results

### 3.1. Overall characteristics

**Figure 3.**
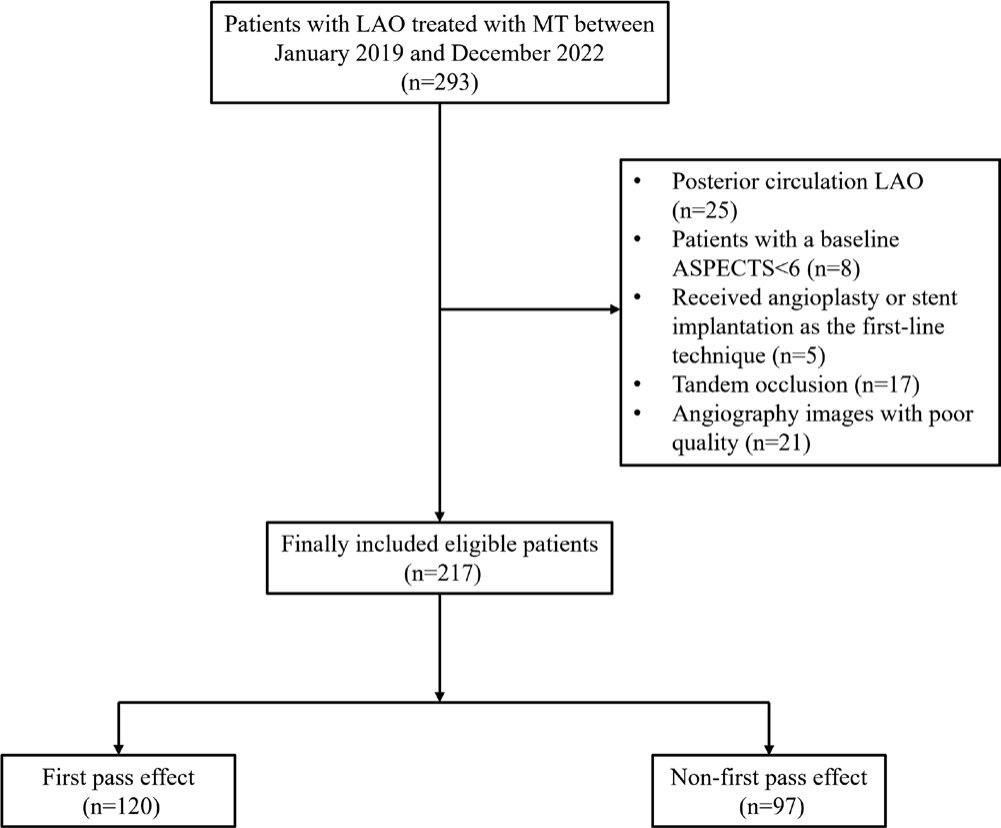
Flow chart of this study. ASPECTS = Alberta Stroke Program Early CT Score, LAO = large artery occlusion, MT = mechanical thrombectomy.

As shown in Figure 3, a total of 217 patients with anterior circulation occlusion were included in the final analysis. In total, 51 patients were excluded from the analysis for the following reasons: baseline ASPECTS < 6 (n = 8); received angioplasty or stent implantation as the first-line technique (n = 5); tandem occlusion (n = 17); and angiography images with poor quality (n = 21). Of the included patients, the mean age was 66.3 ± 14.7 years, and 87 (40.1%) were female. The median NIHSS score on admission was 13 (interquartile range, 10–16), the major occlusion site was 132 (60.8%) with the M1 segment, and the median time from puncture to reperfusion was 59 min (interquartile range, 37–76). Intravenous rt-PA was administered to 70 (32.2%) patients. Among them, combined angiographic technique was conducted in 94 (43.3%) patients.

### 3.2. Comparisons of baseline and procedural characteristics

As shown in Table 1, patients with FPE were had a lower baseline ASPECTS (12 vs 14, *P* = .018), shorter puncture-to-reperfusion time (48 vs 66 min, *P* < .001), and higher thrombolysis rate (39.2% vs 29.7%, *P* = .015) compared with those without FPE. There was a statistically significant difference in first-line thrombectomy strategy between the FPE group and the non-FPE group (*P* < .001). In addition, patients with FPE used the combined angiographic technique prior to MT at a significantly higher rate than those without FPE (69.2% vs 11.3%, *P* < .001). The sensitivity, specificity, positive predictive value, and negative predictive value of the combined angiographic technique in predicting FPE were 69.2%, 88.6%, 88.3%, and 69.9%, respectively (Table 2).

**Table 1:** Comparison of characteristics between patients with and without the achievement of first pass effect.

	FPE (n = 120)	Non-FPE (n = 97)	*P* value
Age (year), mean ± SD	65.9 ± 15.4	66.8 ± 13.9	.947
Female, n (%)	48 (40.0)	39 (40.2)	.975
Comorbid conditions			
Smoking, n (%)	68 (56.7)	58 (59.8)	.643
Hypertension, n (%)	69 (57.5)	60 (61.9)	.516
Diabetes mellitus, n (%)	33 (27.5)	22 (22.7)	.417
Atrial fibrillation, n (%)	39 (32.5)	28 (28.9)	.565
Hyperlipidemia, n (%)	41(34.2)	33 (34.0)	.982
Clinical variables			
Baseline NIHSS, median (IQR)	12 (10–15)	14 (10–16)	.018*
Baseline SBP (mm Hg), mean ± SD	153.0 ± 21.9	152.5 ± 26.3	.881
Baseline DBP (mm Hg), mean ± SD	86.8 ± 14.8	88.3 ± 16.6	.496
Bridging thrombolysis, n (%)	47 (39.2)	23 (23.7)	.015*
Puncture to reperfusion time (min), median (IQR)	48 (25–64)	66 (54–89)	<.001*
Occlusion site, n (%)			.486
ICA terminus	25 (20.8)	15 (15.5)	
M1 segment	69 (57.5)	63 (64.9)	
M2 segment	20 (16.7)	15 (15.5)	
A1 segment	6 (5.0)	7 (7.2)	
First-line thrombectomy strategy, n (%)			<.001*
CA	72 (60.0)	47 (48.5)	
SR	16 (13.3)	14 (14.4)	
CA + SR	32 (26.7)	36 (37.1)	
CAT, n (%)	83 (69.2)	11 (11.3)	<.001*

ASPECTS = Alberta Stroke Program Early CT Score, CA = contact aspiration, CAT = combined angiographic technique, DBP = diastolic blood pressure, FPE = first pass effect, ICA = internal carotid artery, NIHSS = National Institutes of Health Stroke Scale, SBP = systolic blood pressure, SR = stent retriever.

* *P* indicates statistical significance.

**Table 2:** Predictive value of combined angiographic technique for first pass effect.

	AUC	95% CI	*P* value	Sensitivity	Specificity	PPV	NPV
CAT	0.789	0.727–0.851	<.001	0.692	0.886	0.883	0.699

AUC = area under the curve, CAT = combined angiographic technique, NPV = negative predictive value, PPV = positive predictive value.

### 3.3. Multivariate regression analysis of FPE

The associations of baseline characteristics with FPE were determined using binary logistic regression models. Binary logistic regression indicated that the combined angiographic technique (OR 16.931; 95% CI 7.211–39.751; *P < *.001) is independently associated with FPE after adjusting for baseline NIHSS, bridging thrombolysis, puncture to reperfusion time, occlusion site and first-line thrombectomy strategy (Table 3).

**Table 3:** Binary logistic regression analysis for prediction of first pass effect.

	OR	95% CI	*P* value
Baseline NIHSS	0.855	0.772–0.946	.002
Bridging thrombolysis	1.437	0.642–3.217	.378
Occlusion site	1.350	0.923–1.974	.122
Puncture to reperfusion time	0.986	0.974–0.997	.017
First-line thrombectomy	0.518	0.342–0.786	.002
CAT	16.931	7.211–39.751	<.001

CAT = combined angiographic technique, NIHSS = National Institutes of Health Stroke Scale.

## 4. Discussion

The main finding of this study is that intraprocedural CAT could predicts FPE during MT in patients with anterior circulation large artery occlusion with high specificity and positive predictive value.

The concept of the FPE was first introduced by Zaidat et al in 2018 within a large cohort of patients from the North American Solitaire Acute Stroke (NASA) Registry database.^[6]^ The FPE is a technical success metric for MT in the anterior circulation, achieving complete recanalization of the occluded artery with a single pass.^[6]^ The rates of FPE reported in previously published retrospective data varied greatly, from 15% to 40%.^[5,16,17]^ Compared with previous studies, the incidence of FPE in our retrospective study was relatively high, reaching 55.3%. We speculate that it may be attributed to the use of CAT in our study.

To the best of our knowledge, this is the first study to demonstrate that neuro-interventionists could apply what we refer to as combined angiographic technique during endovascular procedure to predicts FPE. For the routine procedure of thrombectomy, neuro-interventionists perform angiography with an intermediate catheter, which can only illuminate the proximal end of the thrombus, but not the distal end. As a result, the specific location and length of the thrombus cannot be determined. The innovation of CAT lies in the fact that angiography is simultaneously performed with an intermediate catheter and micro-catheter. Due to cerebral collateral circulation, the contrast medium follows retrograde blood flow and displays the distal end of the thrombus clearly.

Currently, mainstream thrombectomy methods include contact aspiration (CA) and stent retrieval.^[2]^ The physical interaction between the retriever stent and the thrombus is crucial for the success of MT. This necessitates the precise positioning and selection of an appropriate retriever stent size. By employing the CAT technique, the stent retriever can be accurately positioned in the optimal location with respect to the thrombus, facilitating a secure connection between the thrombus and the stent retriever. This may be beneficial in reducing the likelihood of secondary downstream embolic events and the dislodgment of thrombus fragments. Furthermore, the stent retriever may more effectively capture the thrombus in its entirety, hence ensuring consistent distribution of radial forces along the thrombus during retraction. On the other hand, a stent retriever thrombectomy’s efficacy is also significantly influenced by vascular curvature.^[18]^ For example, if the distal vascular bed is twisted and without a landing area for the stent, the stent retriever may stretch the distal vessel excessively, resulting in bleeding. Moreover, if the thrombus length is short, indicating a low thrombus burden and migration risk, the CA technique can be directly chosen. In summary, due to the clear display of thrombus length and location by CAT, neuro-interventionists can select the appropriate thrombus strategy and optimal material combination, greatly enhancing thrombectomy efficacy and significantly reducing the time from puncture to reperfusion. This might explain why the FPE group had a higher proportion of CA than the non-FPE group in this study (60% vs 48.5%).

In this study, a lower baseline NIHSS score and a higher rate of rt-PA intravenous thrombolysis were observed in the FPE group. One possible explanation may suggest that intravenous thrombolysis led to thrombus fragmentation, which could reduce thrombus burden and increase the success rate of thrombus retrieval. Additionally, a lower baseline NIHSS score may indicate a lower baseline thrombus burden, which may be linked to a higher success rate of thrombectomy. Prior studies have also demonstrated that AIS patients with a high baseline thrombus burden score were more likely to experience successful recanalization following reperfusion treatments.^[19]^

Limitations include the study being a single-center experience and with a relatively small number of patients with anterior circulation ischemic strokes. Second, in this observational study, the choice of CAT and the first-line MT method was determined at the discretion of the neuro-interventionists and was subsequently heterogeneous. Third, information on the caliber of aspiration catheters and the size of retrieval devices was not collected and analyzed, which limits the generalizability of this study. Fourth, techniques included in this study may suffer from biases such as potential technical tips, the operators’ collaboration, and equipment barriers. Fifth, in addition to FPE, symptomatic intracranial hemorrhage (sICH), puncture to recanalization time, and the 3-month mRS score may serve as other outcome evaluation indicators. However, due to the retrospective nature of this study, the aforementioned data were not collected. Finally, the established associations in this study may be impacted by other potential baseline characteristics that are not measured, which are worthy of further investigation.

## 5. Conclusion

Our study provides preliminary data indicating that CAT is associated with FPE in LAO patients following MT, which could assist neuro-interventionists in selecting the appropriate thrombectomy strategy and optimal material combination. Larger prospective studies that are conducted across multiple centers are necessary to validate the safety, efficacy, and generalizability of the CAT technique.

## Author contributions

**Conceptualization:** Peng Wang.

**Data curation:** Danyu Chen, Shufeng Yu.

**Formal analysis:** Danyu Chen.

**Funding acquisition:** Chao Xu, Peng Wang.

**Investigation:** Tianbo Xu.

**Methodology:** Guixing Xu.

**Project administration:** Jiangxian Ying.

**Supervision:** Peng Wang.

**Writing – original draft:** Chao Xu.

**Writing – review & editing:** Peng Wang.
